# Cellular Signaling for Dental Physiological Functions

**DOI:** 10.3390/biom13081177

**Published:** 2023-07-28

**Authors:** Takehito Ouchi, Taneaki Nakagawa

**Affiliations:** 1 Department of Physiology, Tokyo Dental College, Tokyo 101-0061, Japan; 2 Department of Dentistry and Oral Surgery, Keio University School of Medicine, Tokyo 160-8582, Japan

Teeth are unique and multifaceted tissues that are necessary for routine functions, such as crushing food and constructing articulated sounds, as well as for esthetic symbols. Nociceptive responses to external stimuli in the dentin have been detected in the dental pulp and neuron complex [1]. Surrounding tissues, such as the gingiva, play defensive roles against the oral microbiota [2]. Thus, dental and periodontal tissues respond precisely to external stimuli and perform oral functions by cooperating with the surrounding hard and soft tissues.

However, an imbalance between these hard and soft tissues leads to pathological findings in oral tissues, including teeth, thereby resulting in dysfunctional physiological processes. To prevent this phenomenon, intracellular signaling control, intercellular communication, genetic regulation, and functional regenerative medicine based on tissue engineering have been considered [3]. In a previous edition titled “Tissue Regeneration and Physiological Functional Recovery in Dental and Craniofacial Fields”, we discussed the potential of oral cells in tissue regeneration and functional recovery [4]. In the second edition, several studies were discussed and researchers showed the other potentials of oral and cranial stem cells to use in advanced therapeutic strategies.

Lyu et al. studied apical periodontitis caused by bacterial infections as well as reviewed recent studies on it, focusing on the regulatory network of mesenchymal stem cells (MSCs) [5]. Apical periodontitis is a common inflammatory disease of the periapical region of teeth. Regenerating the destroyed periapical alveolar bone and its surrounding periodontal tissues is clinically difficult. These lesions are closely related to pathogen invasion and hyper-reactive immune responses. Simultaneously, protective healing processes occur in teeth, and MSCs play important roles in mediating the immune system and promoting regeneration [5]. Thus, MSCs are a differentiation agent and a core of the immune system.

The functions and properties of cells are regulated by niche microenvironments. The dental pulp is a soft tissue of a cranial neural crest-derived mesenchymal origin enclosed by a rigid mineralized dentin, which ultimately resides in a low-compliance environment [6]. When an inflammatory response is elicited in the dental pulp, internal tissue pressure increases, which mechanically stimulates dental pulp cells, including nerve terminals and odontoblasts [7]. Saito et al. developed co-culture models that mimicked the axon reflex in the dental pulp [8]. Odontoblasts differentiate from dental pulp stem cells and localize to the outermost layer of the dental pulp [9]. Odontoblasts drive physiological, developmental, and pathological tertiary dentin formation and biological defensive dentin formation. Odontoblasts play an essential role in tooth pain sensitivity via signal communication with trigeminal ganglion (TG) neurons as sensory receptor cells [10]. Calcitonin gene-related peptide (CGRP) is a 37-amino-acid and a well-known neuropeptide that is primarily localized to C and Aδ sensory fibers. These fibers show wide innervation throughout the body with great perivascular localization and play a dual role in the functions of sensory and efferent nerves [11]. Previous studies suggested that the activation of CGRP and its receptors plays roles in axon reflex development, leading to the neurogenic inflammation of the dental pulp; however, mechanisms underlying the functions of CGRP and its receptors in dental pulp cells, which determine whether dentin regeneration occurs in response to pulpitis, remain unclear. Furthermore, direct evidence of axon reflex occurrence in the dental pulp via neuropeptide signaling is lacking. Saito et al. determined the role of the CGRP–CGRP receptor axis in axon reflex occurrence by measuring intracellular cyclic adenosine monophosphate (cAMP) ([cAMP]_i_) levels via Gα_s_ protein-coupled CGRP receptor activation in odontoblasts [8]. The authors developed a model that mimicked the effect of increased tissue pressure on inflammatory responses in the dental pulp, and direct mechanical stimulation was applied to TG neurons. The [cAMP]_i_ response from the odontoblasts approximating the stimulated TG neurons was measured using an odontoblast–neuron co-culture system. The authors revealed the functional expression of Gα_s_ protein-coupled CGRP receptors in odontoblasts. CGRP receptor activation increases [cAMP]_i_ levels by the action of adenylyl cyclase. The mechanical stimulation of small-sized CGRP-positive but neurofilament heavy chain-negative TG neurons increases [cAMP]_i_ levels in odontoblasts localized near the stimulated neurons.

These data suggest that the mechanical stimulation of the peptidergic C fiber in TG neurons, which mimics the mechanical stimulation caused by dental pulp inflammation, induces CGRP release after activating the mechanosensitive ion channel. The authors revealed that CGRP increased [cAMP]_i_ levels in odontoblasts by activating CGRP receptors and decreased dentin mineralization. Thus, the CGRP–CGRP receptor axis plays a critical role in regulating dentinogenesis via intercellular communication. These results may provide functional evidence for axon reflexes mediated by the CGRP–CGRP receptor axis in the dental pulp [8].

The adenylyl cyclase signaling pathway, regulated by G-protein-coupled receptors (GPCRs), induces intracellular cAMP signaling in various cells [12,13]. G proteins are composed of α, β, and γ subunits and are mainly divided into Gα_s_, Gα_i_, Gα_q_, and Gα_12/13_. Many receptor conformations lead to various highly specialized downstream signaling cascades. GPCRs induce two major signaling pathways, namely cAMP and phosphatidylinositol signaling. Gα_s_ and Gα_i_ regulate the cAMP-generating enzyme adenylyl cyclase. Gα_s_ activates adenylyl cyclase, whereas Gα_i_ inhibits adenylyl cyclase, thus increasing or decreasing [cAMP]_i_ levels. Diffusible intracellular secondary messenger systems can stimulate or inhibit downstream signaling, thereby exerting further biological effects [14].

Kitayama et al. reported that in the presence of plasma membrane Gα_s_, prostaglandin I2 (IP), 5-hydroxytryptamine 5-HT4 (5-HT4), dopamine D1 (D1), adenosine A2A (A2A), and vasoactive intestinal polypeptide (VIP) receptors, immunoreactivity was observed in human odontoblasts [15]. Furthermore, in the presence of extracellular Ca^2+^, the application of IP, 5-HT4, D1, A2A, and VIP receptor agonists increased intracellular cAMP levels. This increase was inhibited by using an adenylyl cyclase inhibitor and the abovementioned receptor antagonists in a dose-dependent manner. These results suggest that odontoblasts functionally express the G_s_-protein-coupled IP, 5-HT4, D1, A2A, and VIP receptors [15].

Primary cilia are microtubule-based organelles that serve as hubs for transducing various developmental signaling pathways, including Hedgehog, Wnt, fibroblast growth factor, and platelet-derived growth factor signaling [16]. Ciliary dysfunction contributes to diverse disorders collectively known as ciliopathies. Recently, researchers’ interest, especially that of craniofacial biologists, in these syndromes has grown, because many known and putative ciliopathies exhibit severe craniofacial defects [16]. Ciliary signal transduction differs from signaling pathways orchestrated by the cell membrane; however, sometimes, it is complementary or additive. According to these characteristics, the primary cilium is a cellular antenna that operates as a signaling nexus to direct cellular behaviors. Primary cilia are isolated sensory organs, which extend from the surfaces of almost all vertebrate cells, including craniofacial cells. Primary cilia transduce external chemical and physical stimuli to carry out intracellular signaling cascades and simultaneously mediate several well-known signaling pathways. Therefore, primary cilia are the couplers and amplifiers of cell signaling. Primary ciliary dysfunction leads to many diseases and syndromes that directly affect multiple organs, including the face and teeth [17,18,19,20,21]. Previous studies on primary cilia in mineral tissues will be helpful for our understanding of cilia function in craniofacial development and repair.

Collagen type I alpha 1 (Col1A1) and dentin matrix protein 1 (DMP-1) are vital matrix molecules in biomineralization [22,23,24,25]. Arivalagan et al. performed an ingenuity pathway analysis and identified the molecular interaction network of DMP-1 and Col1A1 with transforming growth factor beta receptor II interacting protein-1 (TRIP-1) [26]. TRIP-1 is localized in the mineralized matrices of the bone and dentin [27]. Based on its localization pattern in the bones and teeth, it was suggested that TRIP-1 functioned as a regulatory protein with multiple functions during mineralization. Chen and George reported the in vivo function of TRIP-1 by performing an implantation assay using recombinant TRIP-1 and TRIP-1-overexpressing and -knocked down cells embedded in a three-dimensional biomimetic scaffold [28]. After four weeks, subcutaneous tissues from the TRIP-1-overexpressing cells and scaffolds containing recombinant TRIP-1 showed higher levels of several extracellular matrix (ECM) proteins, such as fibronectin and collagen I. Furthermore, TRIP-1 gene knockdown and/or downregulation decreased fibronectin levels [28]. The characterization of the TRIP-1 structure provided valuable insights into its role in the ECM of calcified matrices. TRIP-1 does not contain a characteristic amino acid signature or show a structure of classical calcium-binding acidic proteins involved in mineralization. The prediction of its function using several bioinformatics tools suggested TRIP-1 as a multifaceted protein and confirmed its mineral-nucleating role in the ECM [26].

Collagen-based materials such as membranes, sponges, matrices, hydrogels, and composite scaffolds have been extensively used in in vivo studies to facilitate bone tissue regeneration. Takayama et al. summarized growth factor (GF) delivery systems that use collagen membranes (CMs) for bone tissue regeneration [29]. Biomaterials and bioactive agents may play a potential role in bone defect repair; thus, they are being explored for developing bone regeneration strategies. Various artificial membranes, particularly CMs, which are extensively used in periodontal therapy and provide an environment that mimics the ECM, play essential roles in promoting bone regeneration. Additionally, numerous GFs have been used in clinical regenerative therapies. However, if the administration of such growth factors is unregulated, their regenerative capacities are not used to the maximum extent, which can lead to side effects. The lack of effective delivery systems and biological carriers limits the use of these growth factors in clinics. Therefore, considering the efficiency of bone regeneration, spaces maintained using both CMs and GFs can synergistically achieve desirable outcomes in bone tissue engineering. Hence, recent studies focused on the possibility of combining CM and GF to effectively promote bone repair. Takayama et al. highlighted the role of GF-containing CMs in bone tissue regeneration and discussed the use of these CMs in preclinical animal models of regeneration. Additionally, authors addressed the potential concerns and suggested future research directions for GF therapy in regenerative science [29].

In this Special Issue, we discussed mineralization systems based on intracellular signaling and tissue engineering using cells, GFs, and scaffolds. We further discussed their potential applications for understanding cranial development and disease models. In the first and second editions, we additionally need to explore the underlying mechanisms of these concepts in vivo, especially in large animal models.
